# Frequency and Prognostic Impact of *ALK* Amplifications and Mutations in the European Neuroblastoma Study Group (SIOPEN) High-Risk Neuroblastoma Trial (HR-NBL1)

**DOI:** 10.1200/JCO.21.00086

**Published:** 2021-06-11

**Authors:** Angela Bellini, Ulrike Pötschger, Virginie Bernard, Eve Lapouble, Sylvain Baulande, Peter F. Ambros, Nathalie Auger, Klaus Beiske, Marie Bernkopf, David R. Betts, Jaydutt Bhalshankar, Nick Bown, Katleen de Preter, Nathalie Clément, Valérie Combaret, Jaime Font de Mora, Sally L. George, Irene Jiménez, Marta Jeison, Barbara Marques, Tommy Martinsson, Katia Mazzocco, Martina Morini, Annick Mühlethaler-Mottet, Rosa Noguera, Gaelle Pierron, Maria Rossing, Sabine Taschner-Mandl, Nadine Van Roy, Ales Vicha, Louis Chesler, Walentyna Balwierz, Victoria Castel, Martin Elliott, Per Kogner, Geneviève Laureys, Roberto Luksch, Josef Malis, Maja Popovic-Beck, Shifra Ash, Olivier Delattre, Dominique Valteau-Couanet, Deborah A. Tweddle, Ruth Ladenstein, Gudrun Schleiermacher

**Affiliations:** ^1^ Equipe SiRIC RTOP Recherche Translationelle en Oncologie Pédiatrique, Institut Curie, Paris, France; ^2^ INSERM U830, Laboratoire de Génétique et Biologie des Cancers, Institut Curie, Paris, France; ^3^ SIREDO: Care, Innovation and Research for Children, Adolescents and Young Adults with Cancer, Institut Curie, Paris, France; ^4^ Department for Studies and Statistics and Integrated Research, Vienna, Austria; ^5^ St Anna Children's Cancer Research Institute, Vienna, Austria; ^6^ Institut Curie Genomics of Excellence (ICGex) Platform, Research Center, Institut Curie, Paris, France; ^7^ Unité de Génétique Somatique, Service de Génétique, Hospital Group, Institut Curie, Paris, France; ^8^ Service de Génétique des tumeurs; Institut Gustave Roussy, Villejuif, France; ^9^ Department of Pathology, Oslo University Hospital, and Medical Faculty, University of Oslo, Oslo, Norway; ^10^ Department of Clinical Genetics, Children's Health Ireland at Crumlin, Dublin, Ireland; ^11^ Northern Genetics Service, The Newcastle upon Tyne Hospitals NHS Foundation Trust, Newcastle upon Tyne, United Kingdom; ^12^ Ghent University, Ghent, Belgium; ^13^ Translational Research Laboratory, Centre Léon Bérard, Lyon, France; ^14^ Instituto de Investigación Sanitaria La Fe, Valencia, Spain; ^15^ Division of Clinical Studies, The Institute of Cancer Research, London, United Kingdom; ^16^ Schneider Children's Medical Center of Israel, Tel Aviv University, Tel Aviv, Israel; ^17^ Departamento de Genética Humana, Instituto Nacional de Saúde Doutor Ricardo Jorge, Lisbon, Portugal; ^18^ Sahlgrenska University Hospital, Göteborg, Sweden; ^19^ Department of Pathology, IRCCS Istituto Giannina Gaslini, Genova, Italy; ^20^ Laboratory of Molecular Biology, IRCCS Istituto Giannina Gaslini, Genova, Italy; ^21^ Pediatric Hematology-Oncology Research Laboratory, Lausanne University Hospital and University of Lausanne, Lausanne, Switzerland; ^22^ Department of Pathology, Medical School, University of Valencia-Incliva Health Research Institute/CIBERONC, Madrid, Spain; ^23^ Center for Genomic Medicine, Rigshospitalet, Copenhagen University Hospital, Copenhagen, Denmark; ^24^ Department of Paediatric Haematology and Oncology, Second Faculty of Medicine, Charles University and University Hospital Motol, Prague, Czech Republic; ^25^ Paediatric Tumour Biology, Division of Clinical Studies, The Institute of Cancer Research, Sutton, United Kingdom; ^26^ Department of Pediatric Oncology and Hematology, Institute of Pediatrics, Jagiellonian University Medical College, Krakow, Poland; ^27^ Clinical and Translational Oncology Research Group, Health Research Institute La Fe, Valencia, Spain; ^28^ Leeds Children's Hospital, Leeds General Infirmary, Leeds, United Kingdom; ^29^ Karolinska University Hospital, Stockholm, Sweden; ^30^ Department of Paediatric Haematology and Oncology, Princess Elisabeth Children's Hospital, Ghent University Hospital, Ghent, Belgium; ^31^ Paediatric Oncology, Fondazione IRCCS, Istituto Nazionale dei Tumori, Milan, Italy; ^32^ Pediatric Hematology-Oncology Unit, Lausanne University Hospital and University of Lausanne, Lausanne, Switzerland; ^33^ Ruth Rappaport Children's Hospital, Rambam Health Care Campus, Haifa, Israel; ^34^ Département d'Oncologie Pédiatrique, Gustave Roussy, Villejuif, France; ^35^ Wolfson Childhood Cancer Research Centre, Newcastle Centre for Cancer, Translational & Clinical Research Institute, Newcastle University, Newcastle upon Tyne, United Kingdom; ^36^ Department for Studies and Statistics and Integrated Research, St Anna Children's Hospital, St Anna Children's Cancer Research Institute, Vienna, Austria; ^37^ Department of Paediatrics, Medical University of Vienna, Vienna, Austria

## Abstract

**PURPOSE:**

In neuroblastoma (NB), the ALK receptor tyrosine kinase can be constitutively activated through activating point mutations or genomic amplification. We studied *ALK* genetic alterations in high-risk (HR) patients on the HR-NBL1/SIOPEN trial to determine their frequency, correlation with clinical parameters, and prognostic impact.

**MATERIALS AND METHODS:**

Diagnostic tumor samples were available from 1,092 HR-NBL1/SIOPEN patients to determine *ALK* amplification status (n = 330), *ALK* mutational profile (n = 191), or both (n = 571).

**RESULTS:**

Genomic *ALK* amplification (*ALK*a) was detected in 4.5% of cases (41 out of 901), all except one with *MYCN* amplification (MNA). *ALK*a was associated with a significantly poorer overall survival (OS) (5-year OS: *ALK*a [n = 41] 28% [95% CI, 15 to 42]; no-*ALK*a [n = 860] 51% [95% CI, 47 to 54], [*P* < .001]), particularly in cases with metastatic disease. *ALK* mutations (*ALK*m) were detected at a clonal level (> 20% mutated allele fraction) in 10% of cases (76 out of 762) and at a subclonal level (mutated allele fraction 0.1%-20%) in 3.9% of patients (30 out of 762), with a strong correlation between the presence of *ALK*m and MNA (*P* < .001). Among 571 cases with known *ALK*a and *ALK*m status, a statistically significant difference in OS was observed between cases with *ALK*a or clonal *ALK*m versus subclonal *ALK*m or no *ALK* alterations (5-year OS: *ALK*a [n = 19], 26% [95% CI, 10 to 47], clonal *ALK*m [n = 65] 33% [95% CI, 21 to 44], subclonal *ALK*m (n = 22) 48% [95% CI, 26 to 67], and no alteration [n = 465], 51% [95% CI, 46 to 55], respectively; *P* = .001). Importantly, in a multivariate model, involvement of more than one metastatic compartment (hazard ratio [HR], 2.87; *P* < .001), *ALK*a (HR, 2.38; *P* = .004), and clonal *ALKm* (HR, 1.77; *P* = .001) were independent predictors of poor outcome.

**CONCLUSION:**

Genetic alterations of *ALK* (clonal mutations and amplifications) in HR-NB are independent predictors of poorer survival. These data provide a rationale for integration of ALK inhibitors in upfront treatment of HR-NB with *ALK* alterations.

## INTRODUCTION

Neuroblastoma (NB), the most frequent solid, extracranial malignancy in children, exhibits wide clinical and genetic heterogeneity. High-risk neuroblastoma (HR-NB), defined as metastatic disease over the age of 12 months or *MYCN*-amplified (MNA) disease at any age, remains associated with long-term survival rates of only 50%.^1^ Current treatment approaches consist of intensive induction chemotherapy, surgical resection of the primary tumor, consolidation with high-dose chemotherapy (HDC), and autologous stem-cell rescue, and for minimal residual disease, isotretinoin in combination with human or mouse chimeric anti-GD_2_ antibody, ch14.18.^2-8^

**CONTEXT**

**Key Objective**
High risk neuroblastoma (HR-NB) is one of the most difficult childhood cancers to cure. This study examined whether the presence of an *ALK* alteration (amplification or mutation) was associated with a poor prognosis in a large patient series treated on the prospective European high-risk neuroblastoma trial (HR-NBL1).
**Knowledge Generated**
We found that *ALK* amplification or clonal mutation was associated with inferior prognosis in patients with HR-NB and both are independent prognostic variables on multivariate analysis. To our knowledge, this is the first study to report the highly prognostic significance of *ALK* amplification in HR-NB.
**Relevance**
As ALK can be targeted therapeutically, this study convincingly argues for the introduction of ALK inhibitors for upfront management of patients with HR-NB with *ALK* aberrations. Importantly, the prognostic significance of *ALK* alterations included a subgroup of trial patients treated with the current standard of care for HR-NB including anti-GD_2_ immunotherapy.

In NB, several recurrent genetic alterations have been described. MNA is a strong biomarker associated with rapid tumor growth.^9^ Other copy-number alterations occur over more extensive chromosome regions, with segmental chromosome alterations being associated with a poor outcome.^10^ Recurrent mutations have been described in the RAS-MAPK pathway, chromatin remodeling genes (*ATRX* and *ARID1A*), and *TERT* rearrangements.^11-14^

Activating anaplastic lymphoma kinase (*ALK*) mutations are the most frequent mutations in NB, occurring in both familial and sporadic cases, with somatically acquired *ALK* mutations (*ALK*m) observed in 6%-12% of sporadic NBs in all risk groups.^15-18^

These *ALK* activating mutations are localized most frequently within the kinase domain at hotspots identified at the F1174, R1275, and F1245 positions, with mutations occurring both at clonal (> 20% mutated allele fraction [MAF]) or subclonal levels (< 20% MAF).^19-23^

*ALK* can also be activated by genomic focal amplification, described in 1%-2% of NBs, almost exclusively with MNA,^17,24^ or, more rarely, following structural rearrangements.^25^ Genetic alterations of *ALK* are associated with poorer survival in the overall NB population.^24,26^ However, their prognostic role in HR-NB has been less well studied.^10,17,24^ Altogether, *ALK* alterations are an important molecular target, given the role of *ALK* as a driver oncogene in NB and its actionability with small molecule therapies.^27-29^

To determine the frequency of *ALK* alterations (mutations and amplifications), their correlation with clinical characteristics, and their prognostic impact in HR-NB, we analyzed a large series of 1,092 diagnostic NB samples from patients on the HR-NBL1/SIOPEN trial.

## MATERIALS AND METHODS

### Patients and Samples

Patients were treated within the HR-NBL1/SIOPEN Protocol (ClinicalTrials.gov: NCT01704716, EudraCT: 2006-001489-17; Protocol [online only]), an international, randomized, multiarm, open-label, phase III trial.^2-5,30,31^ Patients with International Neuroblastoma Staging System stages 2, 3, 4, or 4S with MNA, or International Neuroblastoma Staging System stage 4 without MNA ≥ 12 months of age at diagnosis were eligible for the trial up to 20 years of age. Within the trial, several randomized treatment arms were conducted over different periods (Appendix Fig A1, online only). Induction random assignments included the following: R0—random assignment of prophylactic granulocyte colony-stimulating factor during rapid COJEC induction^31^; R3—comparison of two induction regimens, rapid-COJEC versus modified N7.^32^ HDC was evaluated in the R1 random assignment: busulfan or melphalan versus carboplatin or etoposide or melphalan.^3^ Anti-GD_2_ immunotherapy random assignments during maintenance phase were explored in R2 (2009-2013) and R4 (2014-2017), both comparing dinutuximab beta with oral isotretinoin to dinutuximab beta and subcutaneous interleukin-2 with oral isotretinoin, but with altered schedules.^5,30^ In the interim, dinutuximab beta with oral isotretinoin was the recommended standard.

Patients were enrolled on the HR-NBL1/SIOPEN trial after approval by national regulatory authorities and by national, and institutional, ethical committees or review boards in participating countries. Parents or guardians and patients according to age provided written informed consent for treatment, data collection, and analysis.

**FIG 1.**
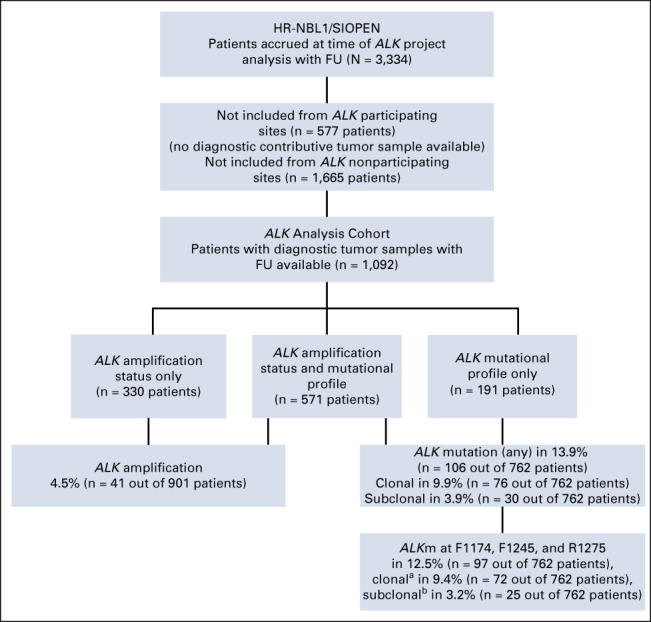
Flow diagram of patient inclusion. A total of 3,334 patients with HR-NB were enrolled in the HR-NBL1 trial from 188 centers. Among these, 2,350 patients were not included in this study, either because no contributive tumor material was available, or because these was no FU data, or both. Thus, 1,092 patients from 132 centers were included in this study. ^a^Clonal level: > 20% MAF. ^b^Subclonal level: MAF 0.1%-20%. FU, follow-up; HR-NB, high-risk neuroblastoma; MAF, mutated allele fraction.

The *ALK* analysis cohort consisted of patients for whom a contributive tumor sample obtained at diagnosis was available in a SIOPEN reference laboratory^33^ for additional molecular analysis with available follow-up data (Fig 1).

*MYCN* status and tumor genomic copy-number profiles were determined in SIOPEN reference laboratories as described previously.^10,33-36^ Samples were required to contain at least 20% tumor cells on pathologic examination.

The *ALK* amplification (*ALK*a) status was evaluated using either fluorescence in situ hybridization and/or multiplex ligation polymerase chain reaction–dependent amplification, array comparative genomic hybridization (aCGH), and/or array single-nucleotide polymorphism according to established guidelines.^10,33,34,37^ *ALK* gene amplification was defined as more than fourfold increase of *ALK* signals in relation to numbers of chromosome 2 by fluorescence in situ hybridization, or as more than 10 copies of the gene estimated by multiplex ligation–dependent amplification, aCGH, or array single-nucleotide polymorphism.

The *ALK* mutational (*ALK*m) status was determined by Sanger sequencing, next-generation sequencing (NGS) techniques (coverage > 80×), targeted deep sequencing (TDS), or a combination of the latter techniques, covering the *ALK* regions of interest (exon 23: chr2:29443647-29443776; exon 24: chr2:29436830-29436935; exon 25: chr2:29432603-29432704; UCSC Genome Browser Home,^38^ hg19) containing the *ALK* mutational hotspots F1174 (exon 23), F1245 (exon 24), and R1275 (exon 25).^20,22^

MAF ≥ 20% were defined as clonal events and MAF < 20% as subclonal events, as reported previously.^20,22^ No correction for tumor cell content was undertaken when reporting MAF. Mutations identified by Sanger sequencing were considered clonal. All detected mutations were validated by a second independent experiment: for clonal events, TDS data were validated by Sanger sequencing, and for subclonal events, NGS or TDS was validated in an independent second experiment.

Standard bioinformatics were used to detect mutations in NGS experiments as previously reported. Mutations in TDS experiments were determined as described previously.^20,22^ In brief, to highlight mutations, in each NB sample, the frequencies of each base at each position of the analyzed regions were compared with those observed in all other samples and controls. This approach enabled the identification of mutations with a statistically significant increase in percentage of a variant base, compared with background noise.

### Statistical Analysis

Event-free survival (EFS) was calculated from diagnosis to the first relapse, progressive disease, secondary malignancy, or death from any cause, or until last patient contact. Overall survival (OS) was calculated from diagnosis to death from any cause, or until the last patient contact. EFS and OS were estimated using the Kaplan-Meier method and compared using the logrank test, and if indicated with pseudo-value regression for 5-year OS.^39-41^ EFS and OS are presented as 5-year point estimates together with 95% CIs using log-log transformation.^41^ To adjust for established risk-factors (age at diagnosis, stage, number of metastatic compartments, and *MYCN* amplification), a Cox proportional hazards regression model was used.

Correlations between patient and disease characteristics and *ALK* genetic alterations were explored using chi-square tests.

To allow for sufficient follow-up time, only patients enrolled until December 31, 2019, were considered. The data cutoff for the final analysis was October 3, 2020. We calculated median follow-up using the inverse Kaplan-Meier estimate. Statistical analysis was performed using SAS (version 9.4).

## RESULTS

Of 3,334 patients enrolled on the HR-NBL1/SIOPEN trial between November 24, 2002, and December 31, 2019, 1,092 patients were included in the *ALK* analysis cohort (Fig 1; Appendix Table A1, online only). Patients were accrued from 132 SIOPEN member institutions or hospitals in 19 countries (Appendix Table A2, online only). Among these 1,092 patients, 81% (889 out of 1,092) were > 18 months of age at diagnosis, 47% (521 out of 1,092) showed MNA, and 88% (966 out of 1,092) had stage 4 disease, with no statistically significant difference in EFS or OS between the *ALK* analysis cohort and the overall HR-NBL1 cohort (Appendix Fig A2, online only).^42^ The median follow-up period was 6.8 years (0.1-17.4 years).

### *ALK* Alterations

Within the ALK cohort, the *ALK*m status was analyzed in 762 patients, the *ALK*a status in 901 cases, with both *ALK*m and *ALK*a studied in 571 patients (Fig 1, Table 1).

**TABLE 1.**
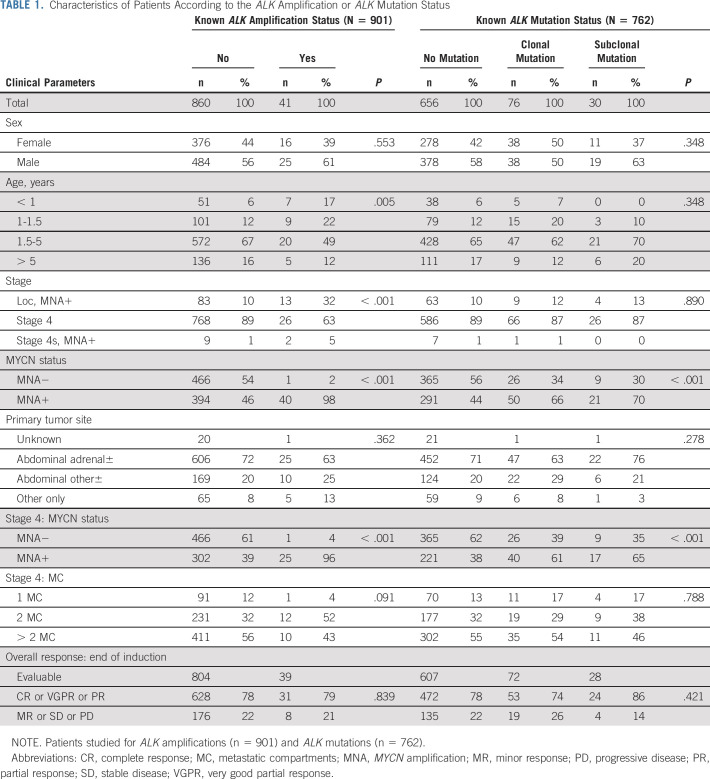
Characteristics of Patients According to the *ALK* Amplification or *ALK* Mutation Status

*ALK* alterations were detected in 146 out of 1,092 patients with *ALK*a occurring in 4.5% (41 out of 901 cases) and *ALK*m in 13.9% (106 out of 762 cases). Only one case showed *ALK*a and a concomitant *ALK* R1275Q mutation with an MAF of 93%, suggesting that the mutated allele is contained in the amplicon (Appendix Fig A3, online only).

### *ALK* Amplification and Correlation With Risk Factors

**FIG 2.**
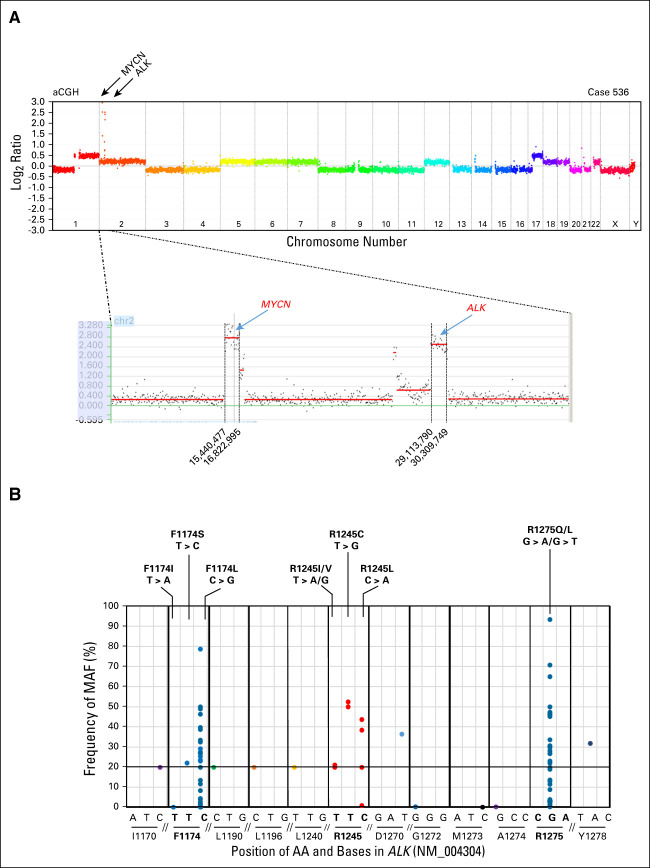
Genetic alterations of *ALK* in patients with HR-NB. (A) Copy-number profile of case 536. Genomic coamplification of *MYCN* and *ALK* is observed on chromosome 2, encompassing the regions between position 15,440,477 and 16,822,999 and between 29,113,790 and 30,309,749 bp (human genome assembly hg19; UCSC Genome Browser Home^38^). (B) Frequency distribution of mutated *ALK* alleles at the studied chromosome regions, encompassing the AA positions F1174, L1190, L1196, R1245, D1270, G1272, M1273, A1274, R1275, and Y1278 detected, in 762 samples. *ALK* mutations involved the common mutational hotspots (F1174, F1245, and R1275) in 12.5% (97 out of 772) of cases, at a clonal level (MAF 20%-93%) in 72 cases, and at a subclonal level (MAF < 20%) in 25 cases. At the F1174 hotspot (chr2: 29,443,695-29,443,697), alterations were observed in 44 cases: 42 cases harbored a mutation leading to the AA change F1174L, one case with F1174I, and one case with F1174S, with MAFs ranging from 0.12% to 78%. At the R1275 hotspot (chr2: 29,432,849-29,430,139), mutations were detected in 43 cases: 38 cases harbored a mutation leading to the AA change R1275Q and five cases with R1275L, with the MAFs ranging from 0.2% to 93%. Ten cases showed *ALK* mutations at the F1245 hotspot (chr2: 29,436,858-29,436,860) within exon 24. Three samples showed the F1245L mutation, three cases carried the F1245C mutation, three showed the F1245I mutation, and one showed mutation F1245V mutation (Fig 1 and Appendix Table A1). Other *ALK* mutations were detected at residues I1170, L1190 (two cases), L1196, D1270, G1272, M1273, A1274, and Y1278 within the explored regions, leading to a nonsynonymous AA change with a predicted functional impact. All these mutations were clonal (MAF > 20%) except for M1273I (MAF 0.2%) and I1170 (MAF 2.8%). AA, amino acid; aCGH, array comparative genomic hybridization; bp, base pair; HR-NB, high-risk neuroblastoma; MAF, mutated allele fraction; UCSC, University of California, Santa Cruz.

High-level genomic amplification of the *ALK* gene was found in 4.5% (41 out of 901) of cases (Fig 2A, Table 1). All but one also had *MNA*. *ALK*a significantly correlated with *MNA* (*P* < .001), non–stage 4 disease (*P* < .001), and age at diagnosis < 18 months (*P* = .005). No correlation between the presence of *ALK*a and response at the end of induction treatment was observed.

**FIG 3.**
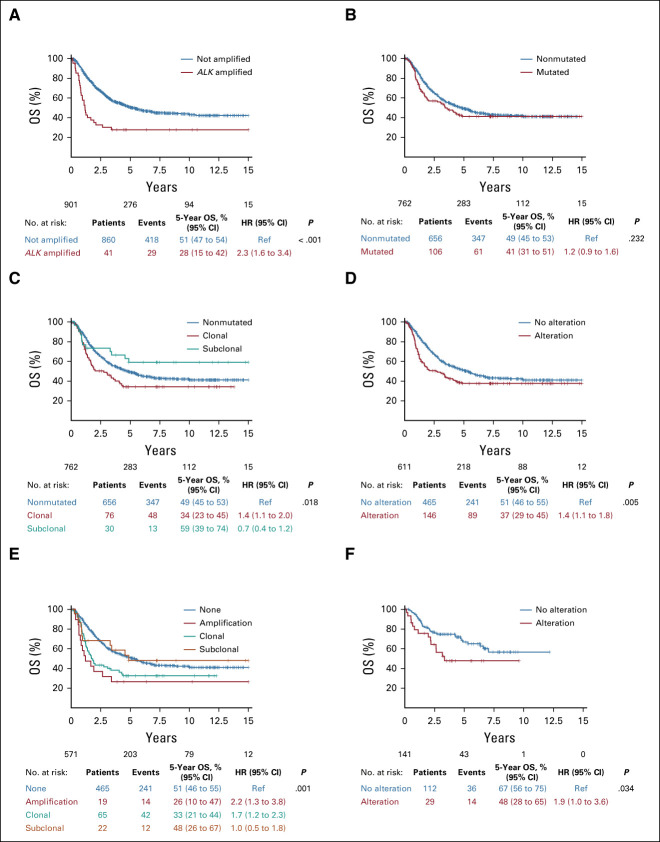
Survival in the *ALK* analysis cohort. (A) OS according to *ALK* amplification status in 901 patients: presence of *ALK* amplification (n = 41), 5-year OS 28% (95% CI, 15 to 42) versus absence of *ALK* amplification (n = 860), 5-year OS 51% (95% CI, 47 to 54); *P* < .0001. (B) OS according to *ALK* mutation status in 762 patients: presence of an *ALK* mutation (n = 106), 5-year OS 41% (95% CI, 31 to 51) versus absence of an *ALK* mutation (n = 656), 5-year OS 49% (95% CI, 45 to 53); *P* = NS. (C) OS according to *ALK* clonal or subclonal mutation status in 762 patients: no mutation (n = 656), 5-year OS 49% (95% CI, 45 to 53); clonal mutations (n = 76), 5-year OS 34% (95% CI, 23 to 45); and subclonal mutations (n = 30), 5-year OS 59% (95% CI, 39 to 74), respectively; *P* = .018. (D) OS according to the presence of any *ALK* alterations in 611 patients with known *ALK* amplification and *ALK* mutation status: presence of an *ALK* alteration (n = 146), 5-year OS 37% (95% CI, 29 to 45); versus absence of *ALK* alterations (n = 465), 5-year OS 51% (95% CI, 46 to 55); *P* = .005. (E) OS according to the type of *ALK* alteration in the cohort of 571 patients with known *ALK* amplification and *ALK* mutation status: no alteration (n = 465), 5-year OS 51% (95% CI, 46 to 55); clonal mutations (n = 65), 5-year OS 33% (95% CI, 21 to 44); subclonal mutations (n = 12), 5-year OS 48% (95% CI, 26 to 67); and *ALK* amplification (n = 19), 5-year OS 26% (95% CI, 10 to 47), respectively; *P* = .001. (F) OS according to *ALK* alterations (*ALK* amplification or clonal *ALK* mutation) in patients who received immunotherapy (n = 141): To evaluate the impact of *ALK* alterations (*ALK* amplification or clonal *ALK* mutation) in patients who received dinutuximab beta, OS was calculated from the start of dinutuximab beta treatment and evaluated using the same approaches as described in the Materials and Methods section. *ALK* alteration (*ALK* amplification or clonal *ALK* mutation, n = 29, 5-year OS 48% [95% CI, 28 to 65]) versus no *ALK* alteration (n = 112) 67% (95% CI, 56 to 75); *P* = .034. Patient details: Appendix Table A3. HR, hazard ratio; NS, not significant; OS, overall survival; ref, reference.

A statistically significant poorer 5-year OS was observed in patients whose tumors harbored *ALK*a (5-year OS: *ALK*a 28% [95% CI, 15 to 42] *v* non-*ALK*a 51% [95% CI, 47 to 54]; *P* < .0001; Fig 3A, Table 2) with a stronger prognostic effect in patients with stage 4 or 4S MNA.

**TABLE 2.**
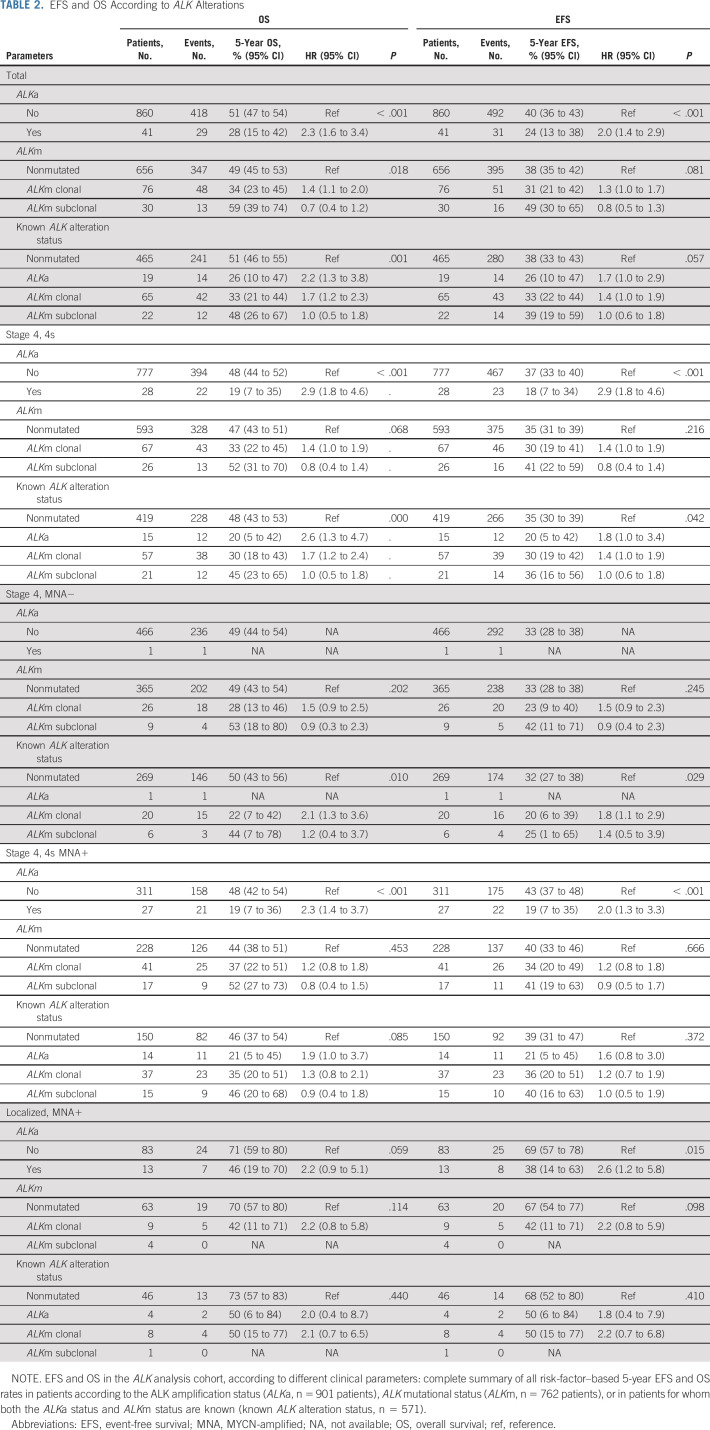
EFS and OS According to *ALK* Alterations

### *ALK* Mutation and Correlation With Risk Factors

*ALK* mutational status was studied in 762 cases by Sanger sequencing (n = 163), by NGS techniques (n = 13), or by TDS (n = 650, including 64 by TDS and Sanger). The biologic data for 52 cases have been reported previously.^22^

Among these, 13.9% (106 out of 762) showed at least one *ALK*m within the explored *ALK* regions of interest, with 10% (76 out of 762) harboring mutations at a clonal level (MAF > 20%) and 3.9% (30 out of 762) at a subclonal level (MAF ≤ 20%): nine cases—MAF 0.1% to < 1%, 10 cases MAF 1% to < 5%, two cases MAF 5% to < 10%, and nine cases MAF 10% to < 20% (Figs 1 and 2B; Table 1).

Concordance between results analyzed by two different techniques was observed in 64 cases with clonal *ALK*m (TDS and Sanger). Subclonal *ALK*m were validated by a second independent TDS experiment, with an excellent correlation of MAF between the two experiments (R2 = 0.9924; *P* < .0001) (Appendix Fig A4, online only).

*ALK*m involved the common mutational hotspots (F1174, F1245, and R1275) in 12.5% (97 out of 762) of cases, comprising 91% (97 out of 106) of all detected *ALK*m (Fig 2B).

Interestingly, three cases harbored two or more distinct mutations. In the first case, both F1174L and F1245L mutations were observed (MAF 2% and 0.8%, respectively). The second case showed three subclonal mutations F1174L, R1275Q, and R1275L (MAF 2.9%, 8.9%, and 2.9%, respectively). A third case harbored a mutation at the F1174 and R1275 hotspots (MAF 27% and 1.3%, respectively).

There were no statistically significant correlations between *ALK*m and stage, age at diagnosis, or localization of the primary tumor (adrenal, abdominal, or other) (Table 1). However, a significant correlation was observed between the presence of an *ALK*m and MNA (*P* < .001), with an enrichment of *ALK*m F1174 in MNA tumors (*P* = .0005). This was also observed when analyzing only stage 4 tumors. No correlation between *ALK*m and response at the end of induction treatment was observed.

No statistically significant difference in outcome was observed between patients harboring any *ALK*m versus none (Fig 3B, Table 2). However, when distinguishing clonal and subclonal mutations, a poorer OS was observed only in patients with clonal *ALK*m, as opposed to subclonal or no mutations (5-year OS, clonal *ALK*m 34% [95% CI, 23 to 45], subclonal *ALK*m 59% [95% CI, 39 to 74], and no *ALK*m 49% [95% CI, 45 to 53]; *P* = .018) (Fig 3C, Table 2).

Patients with metastatic disease (stage 4 or 4S MNA) and a clonal *ALK*m showed a trend toward poorer OS. However, in patients with localized disease, the presence of *ALK*m did not confer poorer survival (Table 2).

### Overall Prognostic Impact of *ALK* Genetic Alterations

To determine the overall prognostic impact of *ALK* genetic alterations, we focused on the subgroup of 571 patients with both known *ALK*a and *ALK*m status. In this subgroup of patients, a statistically significant poorer OS was observed in patients whose tumors harbored any *ALK* alteration (5-year OS, any alteration 37% [95% CI, 29 to 45] *v* no alteration 51% [95% CI, 46 to 55]; *P* = .005; Fig 3D). *ALK*a or clonal *ALK*m were associated with a poorer outcome (5-year OS, *ALK*a 26% [95% CI, 10 to 47], clonal *ALK*m 33% [95% CI, 21 to 44], subclonal *ALK*m 48% [95% CI, 26 to 67], and no *ALK* alteration 51% [95% CI, 46 to 55]; *P* = .001; Fig 3E, Table 2).

Among the subgroup of patients with known *ALK* status, we sought to determine the prognostic impact of *ALK* alterations according to the different treatment arms of HR-NBL1. Indeed, in the HR-NBL01/SIOPEN trial, the introduction of busulfan and melphalan as standard for HDC, and anti-GD_2_ maintenance therapy as a new standard since 2010, has led to significantly improved survival (Appendix Fig A5F, online only).^3-5^ Importantly, when considering patients treated according to the SIOPEN standard with busulfan and melphalan HDC and maintenance immunotherapy, the presence of an *ALK* alteration (*ALK*a or clonal *ALK*m) remained associated with a poorer 5-year OS of 48% (95% CI, 28 to 65), versus no *ALK* alteration 67% (95% CI, 56 to 75); *P* = .03 (Fig 3F, Appendix Table A3, online only), with a trend also observed when taking into account all *ALK*m (clonal and subclonal, *P* = .059).

Based on univariate risk factor exploration of the whole *ALK* analysis cohort (Appendix Fig A5), we developed a Cox model for multivariate analysis including clinical and biologic parameters previously shown to be of prognostic impact (n = 571 patients). Involvement of two or more metastatic compartments (OS: hazard ratio [HR], 2.87 [95% CI, 1.73 to 4.78]; *P* = .001) and the presence of *ALK*a (OS: HR, 2.38 [95% CI, 1.32 to 4.27]; *P* = .004) and clonal *ALK*m (OS: HR, 1.77 [95% CI, 1.25 to 2.49]; *P* = .001) were of independent prognostic significance, whereas *MNA* and age were not (Table 3).

**TABLE 3.**
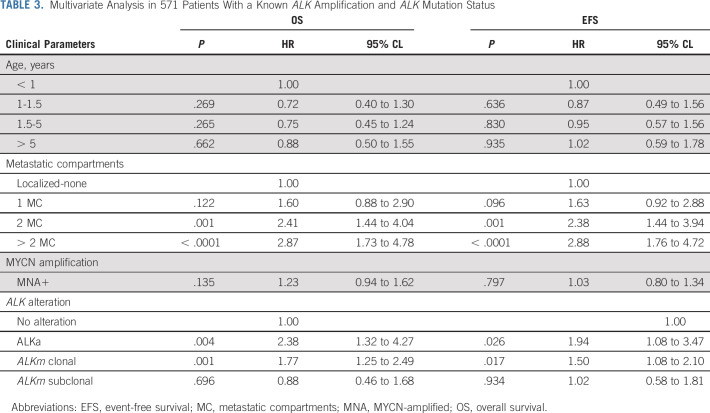
Multivariate Analysis in 571 Patients With a Known *ALK* Amplification and *ALK* Mutation Status

## DISCUSSION

In HR-NB, the identification of prognostic biomarkers is crucial for the development of new treatment approaches. Recent studies have shown that MNA is not associated with poorer outcome among the overall cohort of patients with HR-NB, but the presence of genomic amplifications other than *MYCN* might constitute a poor outcome biomarker.^43^ We now show in this large *ALK* analysis cohort that the presence of *ALK*a or clonal *ALK*m resulted in significantly worse outcome.

Given the oncogenic driver role of *ALK* activation, and the prognostic impact of *ALK*a or clonal *ALK*m, the introduction of frontline ALK-targeted treatment is now strongly supported by the current study. Although early phase clinical trials of first- and second-generation ALK inhibitors showed modest efficacy of the first-generation inhibitor crizotinib in NB with F1174 hotspot mutations being resistant,^44^ third-generation ALK inhibitors such as lorlatinib exhibit improved efficacy alone and when combined with chemotherapy.^28,44-46^ Crizotinib is currently being administered with chemotherapy in a phase III upfront trial for patients with HR-NB with *ALK* alterations (ClinicalTrials.gov: NCT03126916).

Improvements in HR-NB patient survival have been achieved with intensification of HDC and immunotherapy with dinutuximab (ch14.18/Sp02 and ch14.18/CH0),^3-5,7^ and our results highlight the potential of *ALK* inhibition as an attractive upfront precision-medicine strategy in patients with *ALK* alterations to further improve survival. Importantly, in patients reaching the maintenance treatment phase with dinutuximab beta in the HR-NBL1/SIOPEN trial, the presence of an *ALK* alteration was still associated with poorer survival, thus strongly suggesting that integration of *ALK*-targeted therapy is warranted throughout all treatment phases of modern-era HR-NB therapy.

*ALK*a was observed in 4% of NB cases, accounting for approximately 1 out of 3 of *ALK*-activated NB cases. To date, co-occurrence of *ALK* hotspot mutations and genomic amplification has rarely been reported in NB.^17^ In this extensive cohort of patients, one case harboring both *ALK*a and an R1275 *ALK*m was identified. This indicates that these alterations are not fully mutually exclusive, although co-occurrence is extremely rare.

*ALK*m were found in 13.9% of cases at the studied exonic regions harboring known *ALK* mutational hotspots.^17,24^ This is higher than previously reported frequencies of *ALK*m in HR-NB of approximately 10%, most likely as previous reports using Sanger sequencing or standard-resolution NGS approaches.^24,26^ Sanger sensitivity is limited to the detection of MAF > 15%-20%, but in NB, *ALK* mutations with lower MAFs have been reported.^14,19-21^

Ultradeep sequencing used in this analysis has a sensitivity limit of MAF of 0.1%.^19,20^ This approaches the theoretical limit of detection based on the genomic DNA input of 50 ng for one experiment, equivalent to 5,000 diploid genomes.

This study demonstrates that use of higher-resolution techniques enables a higher detection rate of *ALK*m. The MAF distribution indicated a majority of clonal events (76 out of 106 cases). Importantly, clonal *ALK*m were associated with poorer outcome and were of independent prognostic significance, but subclonal events were not. Subclonal events, defined in this study by MAF < 20%, comprised 28% (30 out of 106) of all *ALK*m, with a very low MAF (< 5%) observed in 19 cases.

However, when considering *ALK*m, the OS remains poor in all patient subgroups (5-year OS < 62%). Furthermore, although of different prognostic impact in this study, the biomarker (*ALK* mutation) might not be of distinct predictive impact, and even in patients with subclonal *ALK* mutations, ALK inhibitor treatment might be effective in the targeted cell population. Thus, future upfront trials should consider ALK-targeted treatment based on clinically applicable reliable detection limits (for instance MAF 5% for NGS techniques) rather than the MAF defining prognostic subgroups.

As tumor samples harbored at least 20% tumor cells by pathologic examination, with additional confirmation provided by a dynamic aCGH or SNPa profile in the majority of cases, the observed low MAF is likely to correspond to intratumoral heterogeneity. In NB, intratumor heterogeneity has been reported for MNA and segmental chromosome alterations.^47-49^ The coexistence of *ALK* nonmutated and mutated cells within a single tumor suggests that these different subclones might coexist in an advantageous equilibrium, which might crucially affect the dynamics of cancer progression.^50,51^ Correlation with pathologic findings, single-cell RNA or DNA experiments, and in situ approaches will elucidate how *ALK-*mutated cells are distributed throughout an NB. A higher frequency of *ALK*m at NB relapse has been demonstrated, suggesting clonal evolution of a minor *ALK*-mutated subclone to a dominant *ALK* mutated clone at relapse, but these cases might not represent clinically unfavorable cases initially.^23,52,53^ Further studies focusing on serial blood samples for ctDNA studies will further elucidate clonal evolution, also under targeted therapy.^54^

In HR-NB, mutations in the p53 or RAS-MAPK pathways, including *ALK*, together with telomere maintenance caused by induction of telomerase or ALT (alternative lengthening of telomere) are thought to increase tumor aggressiveness, resulting in even poorer survival among patients with HR-NB.^55,56^ As *MYCN* leads to upregulation of *TERT* expression, MNA associated with any *ALK* alteration might lead to inferior outcome. Cases with *ALK*a show both ALK pathway activation and activation of telomere maintenance through MNA, with a suggested additive effect of these genetic events. The very poor survival of *ALK*a patients is concordant with this observation. However, survival of patients whose tumors harbored *ALK*m and MNA was not different from those without MNA, suggesting that *ALK*m cases constitute a more heterogeneous group with regards to the mechanistic tumor classification.^55^

*ALK*a and *ALK* clonal mutation were both independent predictors of poor outcome in our multivariate Cox model. Notably, the end-of-induction response rate was not associated with *ALK* genetic alterations, suggesting that *ALK-*altered tumor cells are unlikely to be primarily chemotherapy resistant.

In summary, our data contribute to the rationale for future clinical trials introducing ALK-targeted treatment in the frontline setting together with chemotherapy and immunotherapy, and the distinct prognostic impact of different *ALK* alterations (*ALK*a and *ALK*m) needs to be considered.

## Figures and Tables

**FIG A1.**
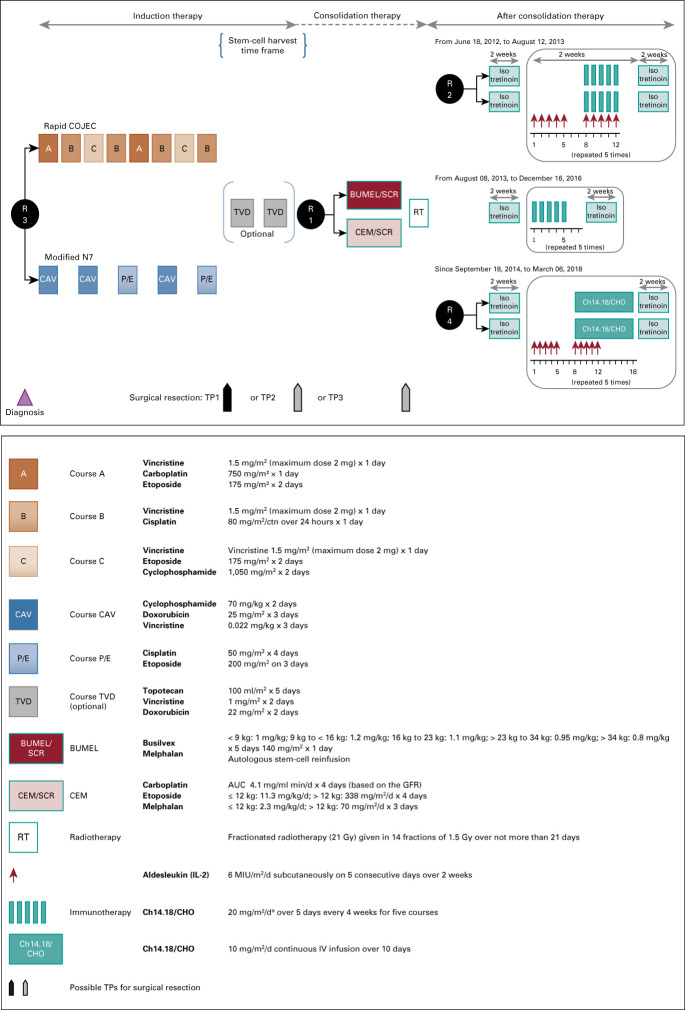
Treatment flowchart of the HR-NBL1 Protocol (ClinicalTrials.gov: NCT01704716, EudraCT: 2006-001489-17) over the whole period. ^a^Infants and children with a body weight below 12 kg will be dosed at 0.67 mg/kg/d. In infants weighing ≤ 5 kg, a further 1/3 dose reduction is advised. AUC, area under the curve; BUMEL, busulfan and melphalan; CAV, cyclophosphamide plus doxorubicin or vincristine; CEM, carboplatin, etoposide, and melphalan; CH14.18/CHO, human-mouse chimeric monoclonal anti-disialoganglioside GD2 antibody ch14.18 produced in Chinese hamster ovary (CHO) cells; COJEC, chemotherapy schedule COJEC defined below; GFR, glomerular filtration rate; IL-2, interleukin-2; IV, intravenous; P or E, cisplatin or etoposide; R1, randomization 1; R2, randomization 2; R3, randomization 3; R4, randomization 4; RT, radiotherapy; SCR, stringent complete response; TP, time period; TVD, topotecan-vincristine-doxorubicin.

**FIG A2.**
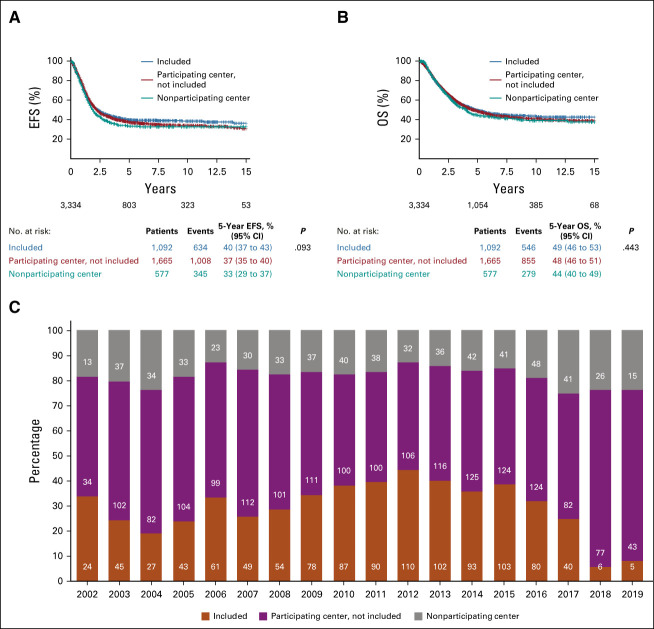
Comparison of patients in the *ALK* analysis cohort and patients not in the *ALK* analysis cohort. (A and B) EFS and OS of the *ALK* analysis cohort and patients not in the *ALK* cohort. (A) No statistically significant difference in EFS and (B) OS was observed between patients included in the ALK analysis cohort (n = 1,092, from 132 centers; red line), patients not included in this study from the same centers (n = 1,665, blue line) and patients not included in this study from centers not participating in this study (n = 577, green line) (5-year EFS: 40% [95% CI, 37 to 43] *v* 37% [95% CI, 35 to 40] *v* 33% [95% CI, 29 to 37]; 5-year OS: 49% [95% CI, 46 to 53] *v* 48% [95% CI, 46 to 51] *v* 44% [95% CI, 40 to 59]; *P* = NS). (C) Recruitment, by year (*x*-axis), in the *ALK* analysis cohort (% of patients: *y*-axis; absolute numbers: in the blue bars). The % and number of patients not included in the ALK analysis cohort from centers participating, and from nonparticipating centers, are indicated in orange and gray, respectively. EFS, event-free survival; NS, not significant; OS, overall survival.

**FIG A3.**
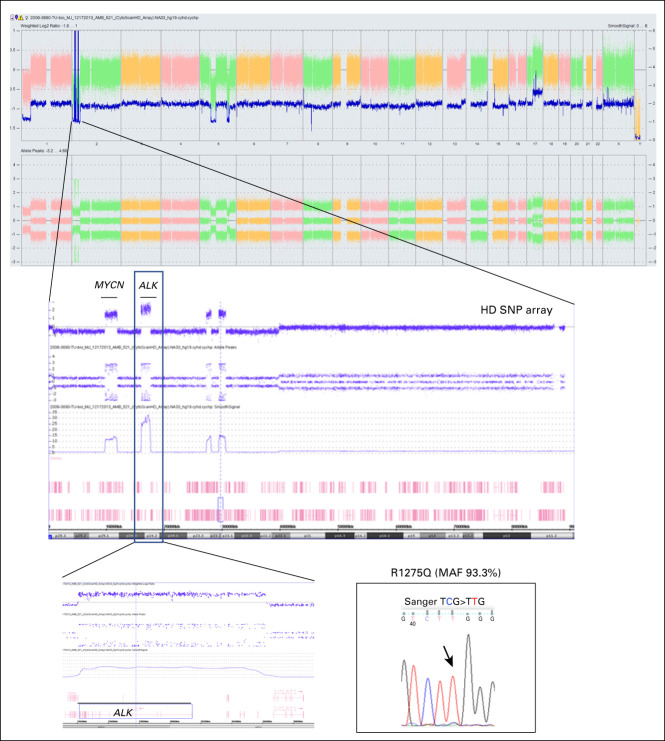
Double event of *ALK* amplification and *ALK* mutation detected in one case (case 15). The SNP array shows an amplified region in chromosome 2 encompassing the *ALK* gene. Sanger sequencing profile shows R1275Q mutation (MAF = 93.3%) in the same case. HD, high definition; MAF, mutated allele fraction; SNP, single-nucleotide polymorphism.

**FIG A4.**
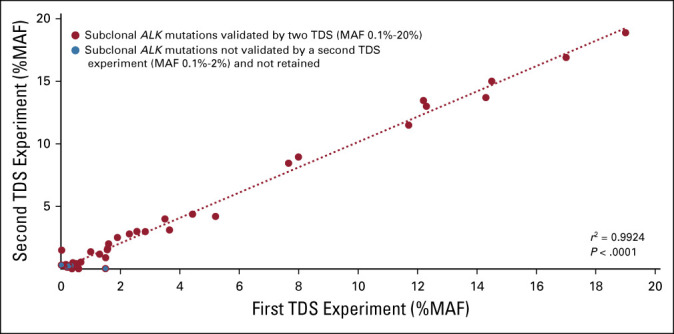
MAF of subclonal *ALK* mutations detected by TDS and confirmed by a second independent TDS experiment. Red spots representing the MAF for each *ALK* mutation are plotted on the *x*-axis (first TDS experiment) and *y*-axis (second TDS experiment), with a strong correlation between the two independent experiments (*r*^2^ = 0.9924, *P* < .0001). Blue spots represent subclonal ALK mutations with a very low MAF (< 0.1%) not confirmed in an independent experiment and not retained in the analysis (n = 6). MAF, mutated allele fraction; TDS, targeted deep sequencing.

**FIG A5.**
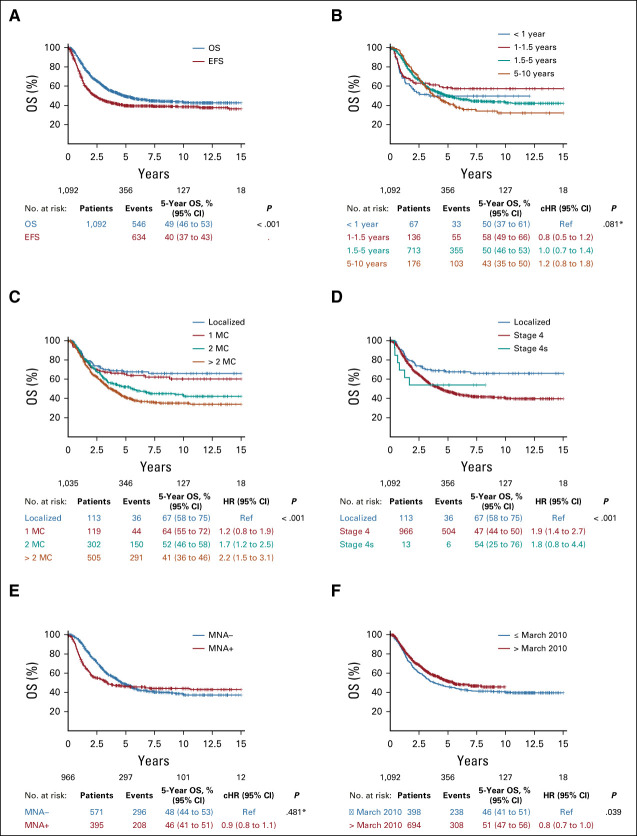
Survival in the *ALK* analysis cohort (n = 1,092 patients) according to known prognostic factors. (A) EFS and OS in the *ALK* analysis cohort population (n = 1,092 patients). Five-year EFS (blue line) 40% (95% CI, 37 to 43); 5-year OS (red line) 49% (95% CI, 46 to 53). (B) OS according to age. Five-year OS in patients < 1 year of age at diagnosis (red line) 50% (95% CI, 37 to 61); in patients 1-1.5 years of age at diagnosis (blue line) 58% (95% CI, 49 to 66); in patients 1.5-5 years of age at diagnosis (green line) 50% (95% CI, 46 to 53); and in patients > 5 years of age at diagnosis (purple line) 43% (95% CI, 35 to 50); *P* = NS (pseudo-value regression). (C) OS according to number of involved MCs. Five-year OS in patients with localized disease (red line) 67% (95% CI, 58 to 75), in patients with involvement of one MC (blue line) 65% (95% CI, 55 to 73), two MCs (green line) 52% (95% CI, 46 to 58), or over two MCs (purple line) 41% (95% CI, 36 to 46); *P* < .001. (D) OS according to stage. Five-year OS in patients with localized disease (red line) 67% (95% CI, 58 to 75), in patients with stage 4 disease (blue line) 47% (95% CI, 44 to 50), or stage 4s disease (green line) 54% (95% CI, 25 to 76); *P* < .001. (E) OS according to *MYCN* amplification in stage 4 disease. Five-year OS in patients with MNA (blue line) 46% (95% CI, 41 to 51), in patients without MNA (red line) 48% (95% CI, 44 to 53), NS (pseudo-value regression). (F) OS according to treatment period, before (< March 2010) or after (> March 2010) the definition of HDC by BUMEL and immunotherapy maintenance as standard treatment. A significant improvement survival because of BUMEL and GD2 standard therapy is observed. Five-year OS in patients having been treated before March 2010 (red line) 46% (95% CI, 41 to 51) versus after March 2010 (blue line) 51% (95% CI, 47 to 56); *P* = .039.^3-5^ BUMEL, busulfan and melphalan; cHR, crude hazard ratio; EFS, event-free survival; HDC, high-dose chemotherapy; HR, hazard ratio; MC, metastatic compartment; MNA, MYCN-amplified; NS, not significant; OS, overall survival; ref, reference.

**TABLE A1.**
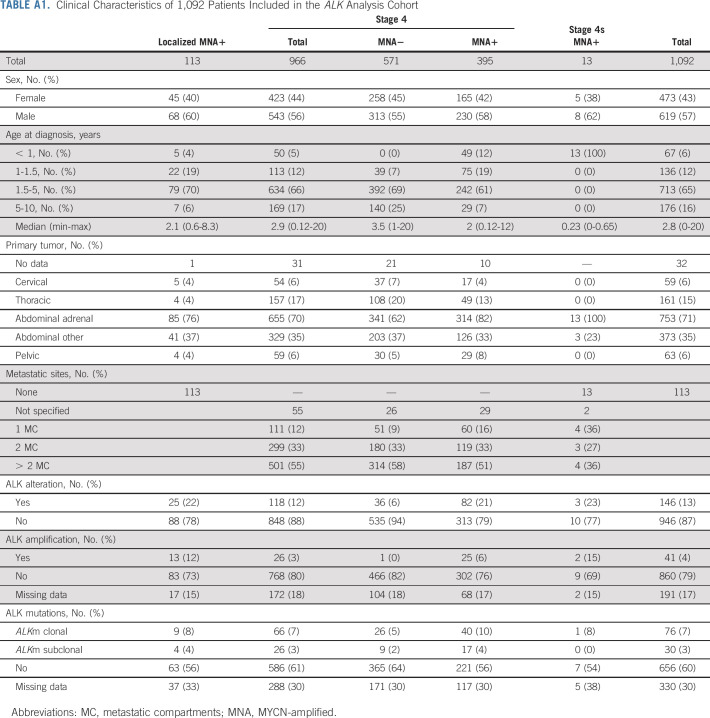
Clinical Characteristics of 1,092 Patients Included in the *ALK* Analysis Cohort

**TABLE A2.**
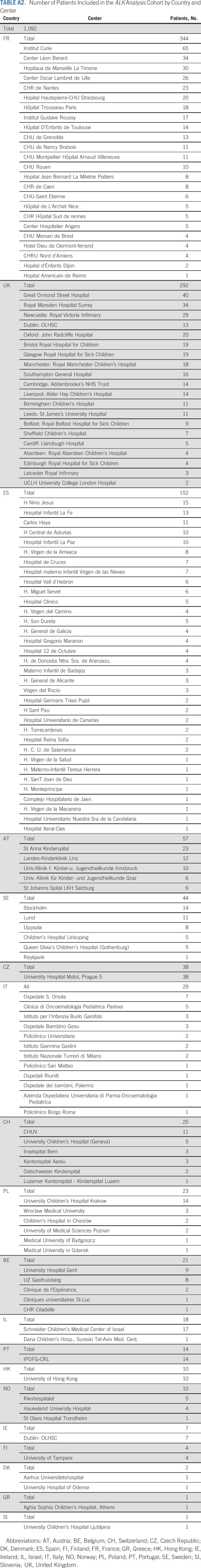
Number of Patients Included in the *ALK* Analysis Cohort by Country and Center

**TABLE A3.**
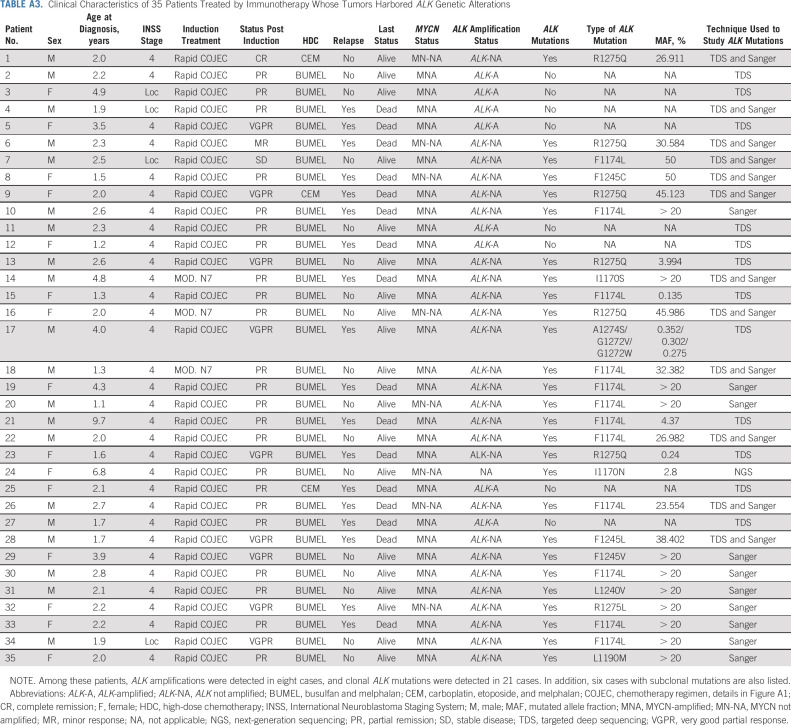
Clinical Characteristics of 35 Patients Treated by Immunotherapy Whose Tumors Harbored *ALK* Genetic Alterations
